# Quantification of echodensities in tuberculous pericardial effusion using fractal geometry: a proof of concept study

**DOI:** 10.1186/1476-7120-10-30

**Published:** 2012-07-28

**Authors:** Mpiko Ntsekhe, Bongani M Mayosi, Tawanda Gumbo

**Affiliations:** 1 Department of Medicine, The Cardiac Clinic, Groote Schuur Hospital and University of Cape Town, Anzio Road, Observatory 7925, Cape Town, South Africa; 2 Office of Global Health, University of Texas Southwestern Medical Centre, Dallas, TX, USA; 3 Department of Medicine, University of Texas Southwestern Medical Centre, Dallas, TX, USA

**Keywords:** Pericardial effusion, Tuberculosis, Fractal dimension, Effusive constrictive pericarditis, Effusive non-constrictive pericarditis

## Abstract

**Background:**

The purpose of this study was to quantify the heterogeneous distribution of echodensities in the pericardial fluid of patients with tuberculous pericarditis using echocardiography and fractal analysis, and to determine whether there were differences in the fractal dimensions of effusive-constrictive and effusive non-constrictive disease.

**Methods:**

We used fractal geometry to quantify the echocardiographic densities in patients who were enrolled in the ***I***nvestigation of the ***M***anagement of ***P***ericarditis ***i***n ***Africa*** (IMPI Africa) Registry. Sub-costal and four chamber images were included in the analysis if a minimum of two clearly identified fibrin strands were present and the quality of the images were of a standard which allowed for accurate measurement of the fractal dimension. The fractal dimension was calculated as follows: D_f_ = limlog *N*(s)/[log (l/s)], where D_f_ is the box counting fractal dimension of the fibrin strand, s is the side length of the box and *N*(s) is the smallest number of boxes of side length s to cover the outline of the object being measured. We compared the fractal dimension of echocardiographic findings in patients with effusive constrictive pericarditis to effusive non-constrictive pericardial effusion using the non-parametric Mann–Whitney test.

**Results:**

Of the 14 echocardiographs from 14 participants that were selected for the study, 42.8% (6/14) of images were subcostal views while 57.1% (8/14) were 4-chamber views. Eight of the patients had tuberculous effusive constrictive pericarditis while 6 had tuberculous effusive non-constrictive pericarditis. The mean fractal dimension D*f* was 1.325 with a standard deviation (SD) of 0.146. The measured fibrin strand dimension exceeded the topological dimension in all the images over the entire range of grid scales with a correlation coefficient (r^2^) greater than 0.8 in the majority. The fractal dimension of echodensities was 1.359 ± 0.199 in effusive constrictive pericarditis compared to 1.330 ± 0.166 in effusive non-constrictive pericarditis (p = 0.595).

**Conclusions:**

The echocardiographic densities in tuberculous pericardial effusion have a fractal geometrical dimension which is similar in pure effusive and effusive constrictive disease.

## Background

**Figure 1:**
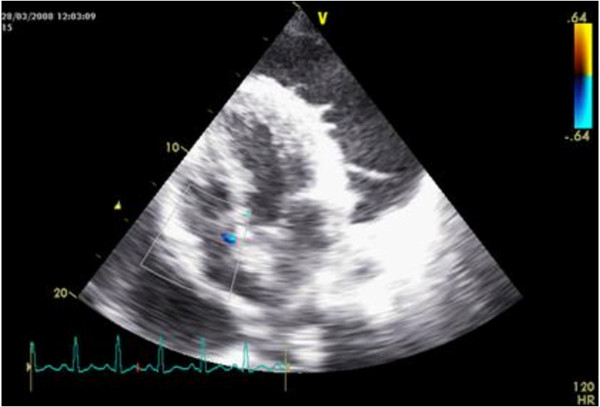
An apical four chamber view of the heart showing frond-like projections from the surface of the left ventricle to pericardial fluid in a patient with tuberculous pericarditis.

Echocardiography is used extensively in patients with pericarditis to identify pericardial effusion, and to look for evidence of cardiac tamponade and constriction [1] It has been proposed that the presence of band-like intrapericardial echocardiographic changes may indicate effusive-constrictive pericarditis [2]. There has also been an attempt to use qualitative changes in echocardiographic densities to diagnose tuberculous pericarditis and to distinguish tuberculous pericarditis from other causes of pericardial disease. Many of the common qualitative descriptions of echo densities found in pericardial effusions include such terms as “fronds”, “snow”, and “strands” [3,4] (Figure 1). These descriptions are subjective, culturally confined, mathematically ambiguous, and are of limited diagnostic value. It is possible that quantitative descriptions of pericardial abnormalities, which have a validated reproducible mathematical method as their basis for their quantification, may overcome these qualitative limitations. Here, we describe a fractal geometry based method to quantify pericardial echo-densities [5]. We applied this method to pericardial tuberculosis, a common problem in Africa, as an example of how this method may be applied to enhance echocardiography based diagnosis.

Pericardial tuberculosis (TB) is a chronic fibrinous pericarditis characterized by granulomatous inflammation and caseous necrosis [6]. Despite being the most common cause of inflammatory pericarditis in the developing world, a non-invasive method of establishing the diagnosis remains elusive, contributing to the high morbidity and mortality associated with the condition [6-8]. As far back as 1893, Sir William Osler reported the presence of “sero-fibrinous exudation with thick fibrin in ridges” resembling “long villous extensions” at autopsy [9]. The echocardiographic equivalent of these pathological findings are fronds-like fibrin strands and the evocatively termed “shaggy coating” of the pericardium [10-12]. The use of 2D echocardiography detected fronds-like fibrin strands as a diagnostic guide for TB pericarditis has been limited by the poor specificity of qualitative methods used in these studies [3,12-14].

The fractal dimension is a measure of the space filling properties of a naturally occurring object [15,16]. Fractal analysis is a method for quantifying naturally occurring objects that are irregular, rough and which cannot be characterized using Euclidean geometry axioms [15,16]. In Euclidean geometry, a point has a dimension of 0, a straight line has a dimension of 1, and a regular planar figure such as a square has a dimension of 2. On the other hand, most pericardial abnormalities detected by echocardiography are irregular and complex and not mere points, straight lines or planar figures. The fractal analytic approach has been used in other settings to quantify the “roughness” or “ruggedness” of objects such as rivers, coasts of seas, leaf veins, the heterogeneity of blood flow, and the clearance of drugs [15,17-19]. This has led to the calculation of dimensions that exceeded the Euclidean topological dimension.

In this proof of concept study we have used fractal analysis as a method to quantify pericardial echocardiographic densities in tuberculous pericarditis. We hypothesized that tuberculosis related fibrin strands are associated with a specific reproducible fractal dimension that was measurable using a simple box counting method. In addition, we investigated if the fractal dimension could be used to differentiate patients with tuberculous effusive constrictive pericarditis from those with tuberculous effusive non-constrictive pericarditis, in which case the fractal dimension may reflect progression from a pure effusive state to an early stage of constrictive pericarditis, in addition to etiology.

## Methods

### Patient selection

This is a sub-study of the Investigation of Management of Pericarditis in Africa (IMPI Africa) prospective registry of patients with tuberculous pericarditis [20]. The Human Research Ethics Committee at the University of Cape Town approved the study (HREC REF: 102/2003). The patients were referred from four community hospitals of Cape Town to Groote Schuur Hospital for investigation of suspected tuberculous pericarditis by means of a diagnostic or therapeutic pericardiocentesis. Consenting adult patients were included if they had all of the following: an echocardiographically confirmed moderate or large effusion [13,21]; evidence of tuberculous etiology; and complete hemodynamic data from a right heart study and intra-pericardial pressure measurements performed pre- and post-pericardiocentesis. The exclusion criteria were evidence of pre-existing structural heart disease, pregnancy and age under eighteen.

### Echocardiography

Prior to pericardiocentesis all patients were evaluated by 2D echocardiography (GE Vivid *i* portable machine with 5.0 MHz phased-array transducer) Sub-costal and four chamber views were preselected as being optimal for fibrin strand analysis following an internal pilot study to assess the impact of the views on the fractal dimension. In the pilot study there was no significant or systematic difference in the fractal dimensions obtained by using either of these views. Echocardiographic images were included in the analysis if a minimum of two clearly identified fibrin strands were present and the quality of the images were of a standard which allowed for accurate measurement of the fractal dimension. The image with the highest quality and clearest definition was chosen for each patient. After selection of the best quality image it was saved five times in a tagged image file format (TIFF). For this proof of concept fractal dimension study the first 14 participants with echocardiographs that allowed for adequate fractal analysis were selected.

### Diagnosis of tuberculosis and effusive-constrictive pericarditis

The etiology was accepted as tuberculous if the pericardial fluid was an inflammatory exudate with: a) direct microbiological evidence as indicated by positive microscopy, culture or polymerase chain reaction based assay for *Mycobacterium tuberculosis;* or b) either an interferon gamma IFN-γ>50 pg/L or an adenosine deaminase (ADA) >40 IU in pericardial fluid [7]. Criteria for a diagnosis of effusive constrictive pericarditis included failure of the right atrial pressure to fall below 10 mmHg or by 50% after the intra-pericardial pressure had normalized [22].

### Fractal dimension calculation and statistical analysis

Each digital photograph was exported as encapsulated postscripts into the Microsoft PowerPoint program. Five grids of cells (boxes) of side length 2 s, s, s/2, s/4, s/8 and s/16, where s = 7 mm, (or series s/2^m^, where m is a an integer) were created using Adobe Illustrator version CS4. Each grid was imported into PowerPoint and embedded within the five separate images so that there were 15 identical images of the same size that had five embedded grids of descending cell size for each original image. The number of boxes *N* inside which at least one point was covered by some component of the identified fibrin strands was then counted manually by a one author (MN) and recounted by a second author (TG) for quality control. The fractal dimension was calculated as follows [5,15]:

(1) $$D_{\text{f}}=\text{limlog}N\left(\text{s}\right)/\left[\text{log }\left(\text{l}/\text{s}\right)\right]$$

Where *D*_f_ is the box counting fractal dimension of the fibrin strand, s is the side length of the box and *N(*s) is the smallest number of boxes of side length s to cover the outline of the object being measured. Because the limit zero cannot be applied to natural objects, the dimension was calculated by:

(2) $$D_{\text{f}}=d$$

Where *d* is the slope of the graph of logarithm [log] (*N*(s)) against log (l/s).

The fractal dimension was measured five times for each fibrin strand-containing image. For some of the images, no additional information was gained by increasing the box size beyond 7 mm^2^, i.e., as *s* increased the log of s remained constant. Log-log graphs of the side of the length of the square cells within each grid were plotted against the number of cells.

Linear regression of the log-log function was then utilized to calculate the slope. The dimension was calculated as “1 + slope.” The correlation coefficient (r^2^) for the linear regression was also calculated. A second method was used to check the validity of the method, for box sizes where m ≥ 1 in the series s/2^m^. In this method, the fractal dimension was calculated as = (log [number of boxes for s/2^m+1^/number of boxes for s/2^m^])/log 2. An average was calculated for each successive pair of boxes. The Hausodorff-Bescovitch (fractal) dimension calculated based on each these two methods had to be identical. We compared the fractal dimension of echocardiographic findings in patients with effusive constrictive pericarditis to effusive non-constrictive pericardial effusion using the non-parametric Mann–Whitney test.

## Results

Of the echocardiographs acquired from the 14 participants 14 images were deemed to be of high enough clarity and quantity for use in the study. 42.8% (6/14) of the images were from the subcostal view and 57.1% (8/14) were from the 4 chamber view. Eight of the patients had tuberculous effusive constrictive pericarditis while 6 had tuberculous effusive non-constrictive pericarditis. The clinical characteristics of the 14 participants are shown in Table 1. All 14 participants were Africans with a median age of 31 (Interquartile range 28–34) years. Six of them (42.8%) were women and 10 (71.4%) were HIV infected. None of these demographic variables differed significantly between patients with and without tuberculous effusive constrictive pericarditis and only the clinical features that define the effusive constrictive phenotype differed between the two groups.

**Table 1:** Clinical characteristics of the participants who were included in the study

**Variable**	**Number of patients: 14**
Age median (IQR)	39 (29–53)
Males, number (%)	5 (35.7%)
HIV infected (%)	11 (78.6%)
Effusive constrictive pericarditis number (%)	7 (50%)
Pericardial tamponade number (%)	8 (57.1%)
Pericardial fluid volume in liters	1,086 ± 0.466
Culture positive (%)	9 (64.3%)
Polymerase chain reaction positive (%)	6 (42.8%)
Elevated ADA or IFN-γ - number (%)	14 (100%)
Pericardial fluid protein in g/L (range)	64 (range)
Pericardial fluid lactate dehydrogenase in units/dL (range)	1,479 (range)

**Figure 2:**
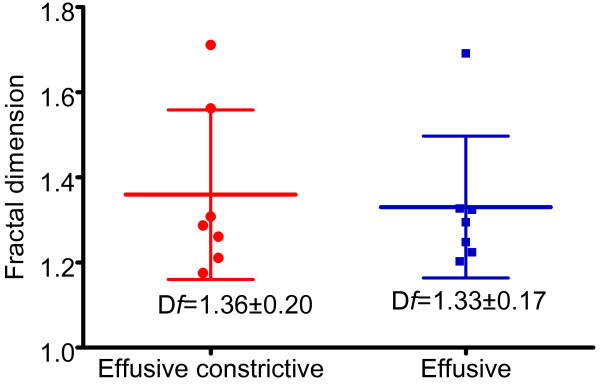
A comparison of the fractal dimension of patients with effusive-constrictive pericarditis versus those with effusive non-constrictive pericarditis.

The fractal dimensions calculated for each of the 14 patients are shown in Table 2. The mean fractal dimension *D*_f_ was 1.325 with a standard deviation (SD) of 0.146. The measured fibrin strand dimension exceeded the topological dimension in all the images over the entire range of grid scales with a correlation coefficient (r^2^) greater than 0.8 in the majority (Table 1). The definition of fractal is a set in which the Hausdoff-Bescovitch dimension exceeds the topological dimension. In this case, in all patients, *D*f exceeded the topological dimension of a line, which is 1. The fractal dimension was 1.359 ± 0.199 in effusive constrictive pericarditis compared to 1.33 ± 0.166 in effusive non-constrictive pericarditis (p = 1.00) (Figure 2). Thus the fractal dimensions were similar in both syndromes of tuberculous pericarditis.

**Table 2:** **The echocardiographic view, fractal dimension, r**^**2**^**of tuberculous pericardial effusion**

**Patient study #**	**Echocardiographic view**	**Fractal dimension**	**R**^**2**^
70	Subcostal	1.248	.972
76	4 chamber	1.2028	.677
78	4 chamber	1.3269	.9484
87	4 chamber	1.3239	.804
88	4 chamber	1.711	.7067
94	4 chamber	1.3081	.8596
95	4 chamber	1.2607	.784
96	4 chamber	1.5622	.8797
101	Subcostal	1.2107	.8966
110	4 chamber	1.6916	.9842
112	Subcostal	1.1754	.877
114	Subcostal	1.2872	.8921
120	4 chamber	1.2948	.9744
123	Subcostal	1.224§	.9939

## Discussion

To the best of our knowledge, we report the first study in which simple and reproducible steps have been applied to quantify the fractal dimension of echocardiographic pericardial effusion. This experiment extends the use of fractal geometry and fractal dimensions to pericardial disease and specifically tuberculous pericarditis. We have demonstrated in this small study that fibrin strands like many naturally occurring objects are a fractal structure and are therefore quantifiable by fractal geometry and dimensions.

Since the recognition that fibrinous pericarditis is associated with various echogenic abnormalities on 2D echocardiography [11], there have been multiple attempts to describe, quantify and utilize these echo abnormalities diagnostically [3,23-25] These attempts have been limited to qualitative descriptive methods. However in the natural sciences, particularly anatomy and pharmacology, the usefulness of fractal geometry in quantifying and describing dimensions is well established [26,27]. Examples include the use of fractal dimension in the description of of neurones [28], motor nerve terminals [29,30], colonies of bacteria [31], the patterns of the cerebral cortex, [32] and antibiotic clearance from patients [19].

We were however unable to demonstrate that the fibrin strand fractal dimension could be used to distinguish effusive constrictive pericarditis from effusive non-constrictive pericarditis. There are however a number of limitations of this study which suggest that this latter result was not unexpected. The first and most important reason is the small number of quality images of the pericardium available to adequately compare the phenotypes. Given the dynamic fluid nature of the fibrin strands and the difficulty capturing adequate still images, future studies addressing this issue will have to consider acquiring more images, with longer cine loops and should consider integrating many more views than the two used in this study.

The fractal dimension of 1.325 for tuberculous pericardial effusion is in itself as interesting as it is famous; it is the fractal dimension of the Apollonian gasket or Leibniz packing [33]. This strongly suggests that the echodensities seen in tuberculous pericarditis are a generalization of inversion transformation, and likely a result of simple iterative process. While this does not reveal the identity of the pathophysiologic process that leads to this lesion, it gives a hint at the mathematical models that can be used to describe the process. We speculate that the fractal dimensions will therefore correspond to specific iterative or repetitive processes, each one linked to specific etiological processes. This study, which has established the fractal dimension of chronic pericarditis, thus lays the basis for a comparative study of the fractal dimension of pericarditis due to different causes such as malignancy and autoimmune disease.

## Conclusion

These results demonstrate that the measured fractal dimension of fibrin strands exceeds the Euclidian geometrical dimension over the entire range of grid scales used. This fulfills the mathematical definition of a fractal structure. It is therefore appropriate to conclude that fractal dimension is the most useful and reproducible tool to quantify this naturally occurring echogenic material in patients with tuberculous pericardial effusion. This proof of principle study opens the possibility for measuring the fractal dimension of other forms of pericarditis, and possibly lay the basis for quantitative definition of different forms of pericarditis using echocardiography.

## Competing interests

The authors declare that they have no competing interests.

## Authors’ contributions

TG conceived the idea for this study. TG, MN and BM designed the study. MN collected the data which he analyzed together with TG. MN wrote the first draft of the manuscript, which was revised by BM and TG. All authors read and approved the final manuscript.

## References

[B1] Wann S Passen E Echocardiography in Pericardial Disease J Am Soc Echocardiogr 2008 21 1 7 13 18165123 10.1016/j.echo.2007.11.003

[B2] Martin R Bowden R Filly K Popp R Intrapericardial abnormalities in patients with pericardial effusion. Findings by two-dimensional echocardiography Circulation 1980 61 3 568 572 7353247 10.1161/01.cir.61.3.568

[B3] George S Salama AL Uthaman B Cherian G Echocardiography in differentiating tuberculous from chronic idiopathic pericardial effusion Heart 2004 90 11 1338 1339 15486140 10.1136/hrt.2003.020081 PMC1768544

[B4] Ku CS Chiou KR Lin SL Liu CP Chaing HT Echocardiographic features of tuberculous pericarditis J Chin Med Assoc 2003 66 10 613 616 14703279

[B5] Falconer K Fractal Geometry: Mathematical Foundations and Applications 1990 Chichester: John Wiley & Sons Ltd

[B6] Mayosi BM Burgess LJ Doubell AF Tuberculous Pericarditis Circulation 2005 112 23 3608 3616 16330703 10.1161/CIRCULATIONAHA.105.543066

[B7] Syed FF Mayosi BM A modern approach to tuberculous pericarditis Prog Cardiovasc Dis 2007 50 3 218 236 17976506 10.1016/j.pcad.2007.03.002

[B8] Mayosi BM Wiysonge CS Ntsekhe M Gumedze F Volmink JA Maartens G Aje A Thomas BM Thomas KM Awotedu AA Mortality in patients treated for tuberculous pericarditis in sub-Saharan Africa S Afr Med J 2008 98 1 36 40 18270639

[B9] Osler W Tuberculous Pericarditis American Journal of the Medical Sciences 1893 105 1 20 36

[B10] Hinds SW Reisner SA Amico AF Meltzer RS Diagnosis of pericardial abnormalities by 2D-echo: A pathology-echocardiography correlation in 85 patients Am Hear J 1992 123 1 143 150 10.1016/0002-8703(92)90758-n 1729817

[B11] Martin RP Bowden R Filly K Popp RL Intrapericardial abnormalities in patients with pericardial effusion Findings by two-dimensional echocardiography. Circulation 1980 61 3 568 572 10.1161/01.cir.61.3.568 7353247

[B12] Chia BL Choo M Tan A Ee B Echocardiographic abnormalities in tuberculous pericardial effusion Am Heart J 1984 107 5 Pt 1 1034 1035 6720508 10.1016/0002-8703(84)90850-0

[B13] Maisch B Seferovic PM Ristic AD Erbel R Rienmuller R Adler Y Tomkowski WZ Thiene G Yacoub MH Guidelines on the diagnosis and management of pericardial diseases executive summary; The Task force on the diagnosis and management of pericardial diseases of the European society of cardiology Eur Heart J 2004 25 7 587 610 15120056 10.1016/j.ehj.2004.02.002

[B14] Barbarin V Xing Z Delos M Lison D Huaux F Pulmonary overexpression of IL-10 augments lung fibrosis and Th2 responses induced by silica particles American Journal of Physiology - Lung Cellular and Molecular Physiology 2005 288 5 L841 L848 15608148 10.1152/ajplung.00329.2004

[B15] Mandelbrot BB The Fractal Geometry of Nature 1983 2 New York

[B16] Roca J de Rozas JM D Manito N Garcia J Rivera I Bosch I Pericardiocentesis: usefulness in the routine determination of intrapericardial pressure Rev Esp Cardiol 1989 42 2 98 104 2781108

[B17] Glenny RW Robertson HT Fractal properties of pulmonary blood flow: characterization of spatial heterogeneity J Appl Physiol 1990 69 2 532 545 2228863 10.1152/jappl.1990.69.2.532

[B18] Mandelbrot BB Stochastic models for the Earth's relief, the shape and the fractal dimension of the coastlines, and the number-area rule for islands Proc Natl Acad Sci U S A 1975 72 10 3825 3828 16578734 10.1073/pnas.72.10.3825 PMC433088

[B19] Hall RG Swancutt MA Gumbo T Fractal geometry and the pharmacometrics of micafungin in overweight, obese, and extremely obese people Antimicrob Agents Chemother 2011 55 11 5107 5112 21876061 10.1128/AAC.05193-11 PMC3195005

[B20] Mayosi BM Wiysonge CS Ntsekhe M Volmink JA Gumedze F Maartens G Aje A Thomas BM Thomas KM Awotedu AA Clinical characteristics and initial management of patients with tuberculous pericarditis in the HIV era: the Investigation of the Management of Pericarditis in Africa (IMPI Africa) registry BMC Infect Dis 2006 6 2 16396690 10.1186/1471-2334-6-2 PMC1352368

[B21] Maisch B Ristic AD Practical aspects of the management of pericardial disease Heart 2003 89 9 1096 1103 12923044 10.1136/heart.89.9.1096 PMC1767862

[B22] Sagrista-Sauleda J Angel J Sanchez A Permanyer-Miralda G Soler-Soler J Effusive-constrictive pericarditis N Engl J Med 2004 350 5 469 475 14749455 10.1056/NEJMoa035630

[B23] Kim SH Song JM Jung IH Kim MJ Kang DH Song JK Initial echocardiographic characteristics of pericardial effusion determine the pericardial complications Int J Cardiol 2008 10.1016/j.ijcard.2008.04.033 18614247

[B24] Alio-Bosch J Candell-Riera J Monge-Rangel L Soler-Soler J Intrapericardial echocardiographic images and cardiac constriction Am Heart J 1991 121 1 Pt 1 207 208 1985368 10.1016/0002-8703(91)90980-v

[B25] Liu PY Li YH Tsai WC Tsai LM Chao TH Yung YJ Chen JH Usefulness of echocardiographic intrapericardial abnormalities in the diagnosis of tuberculous pericardial effusion Am J Cardiol 2001 87 9 1133 1135 A1110 11348622 10.1016/s0002-9149(01)01481-3

[B26] Maisel AS Krishnaswamy P Nowak RM McCord J Hollander JE Duc P Omland T Storrow AB Abraham WT Wu AH Rapid measurement of B-type natriuretic peptide in the emergency diagnosis of heart failure N Engl J Med 2002 347 3 161 167 12124404 10.1056/NEJMoa020233

[B27] Cross SS Cotton DW The fractal dimension may be a useful morphometric discriminant in histopathology J Pathol 1992 166 4 409 411 1517895 10.1002/path.1711660414

[B28] Takeda T Ishikawa A Ohtomo K Kobayashi Y Matsuoka T Fractal dimension of dendritic tree of cerebellar Purkinje cell during onto- and phylogenetic development Neurosci Res 1992 13 1 19 31 1314350 10.1016/0168-0102(92)90031-7

[B29] Tomas J Santafe M Fenoll R Mayayo E Batlle J Lanuza A Piera V Pattern of arborization of the motor nerve terminals in the fast and slow mammalian muscles Biol Cell 1992 74 3 299 305 1628112 10.1016/0248-4900(92)90041-x

[B30] Reichenbach A Siegel A Senitz D Smith TG A comparative fractal analysis of various mammalian astroglial cell types NeuroImage 1992 1 1 69 77 9343558 10.1016/1053-8119(92)90008-b

[B31] Obert M Pfeifer P Sernetz M Microbial growth patterns described by fractal geometry J Bacteriol 1990 172 3 1180 1185 2106504 10.1128/jb.172.3.1180-1185.1990 PMC208582

[B32] Cook MJ Free SL Manford MR Fish DR Shorvon SD Stevens JM Fractal description of cerebral cortical patterns in frontal lobe epilepsy Eur Neurol 1995 35 6 327 335 8591799 10.1159/000117155

[B33] Mandelbrot BB The fractal geometry of nature 1982 W.H. Freeman and Company

